# A Fungal Diterpene Synthase Is Responsible for Sterol Biosynthesis for Growth

**DOI:** 10.3389/fmicb.2020.01426

**Published:** 2020-07-10

**Authors:** Yanjie Liu, Anqing Duan, Longfei Chen, Dan Wang, Qiaohong Xie, Biyun Xiang, Yamin Lin, Xiaoran Hao, Xudong Zhu

**Affiliations:** ^1^Beijing Key Laboratory of Genetic Engineering Drug and Biotechnology, Institute of Biochemistry and Biotechnology, College of Life Sciences, Beijing Normal University, Beijing, China; ^2^Zhejiang Medicine Co., Ltd., Zhejiang, China; ^3^Department of Microbiology, College of Life Sciences, Nankai University, Tianjin, China

**Keywords:** *Pestalotiopsis microspora*, diterpene synthase, MVA genes, sterol biosynthesis, polyketide

## Abstract

A conserved open reading frame, *dps*, is described in *Pestalotiopsis microspora*, sharing a remarkable similarity with fungal diterpene synthases whose function is less studied. Loss-of-function approach manifested that *dps* was necessary for the growth and the development of the fungus. A deletion strain, *dps*Δ, showed a fundamental retardation in growth, which could deliberately be restored by the addition of exogenous sterols to the media. Gas chromatography–mass spectrometry analysis confirmed the loss of the ability to produce certain sterols. Thus, the tolerance and the resistance of *dps*Δ to several stress conditions were impaired. Secondary metabolites, such as the polyketide derivative dibenzodioxocinones, were significantly diminished. At the molecular level, the deletion of *dps* even affected the expression of genes in the mevalonate pathway. This report adds knowledge about fungal diterpene synthases in *Pestalitiopsis microspora*.

## Introduction

Terpenoids are known as one of the most abundant natural products (Ruan et al., 2019). In fungi, terpenoids are usually synthesized from two C_5_ units, isopentenyl pyrophosphate (IPP) and dimethylallyl pyrophosphate (DMAPP) (Li et al., 2015). These two units were condensed *via* the mevalonate (MVA) pathway and undergo a cyclization step to form terpenoid precursors by various terpene synthases, e.g., monoterpene synthase (Lange, 2015), sesquiterpene synthase (Wang et al., 2016), diterpene synthase, triterpene synthase (Dhingra and Cramer, 2017), and even taxadiene synthase (Bian et al., 2017). Due to bioactivities, many of them have been used in diverse fields, such as medicinal chemistry (Austin et al., 1988), food additives (Ravichandran et al., 2018), and biofuels (Wu et al., 2017). Acquiring terpenoids at the industrial level suffers difficulties, e.g., low content in natural materials. Fermentation by microorganisms is an attractive strategy to obtain these natural products, owing to its large scale and comparatively lower cost if compared to other methods, i.e., extraction from raw material or plant cell culture (Gauvin et al., 2000; Kobayashi and Shigemori, 2002). Many fungi have been reportedly used in the production of terpenoids, i.e., pinene, limonene, camphor, and likely artemisinin (Wu et al., 2017).

**FIGURE 1:**
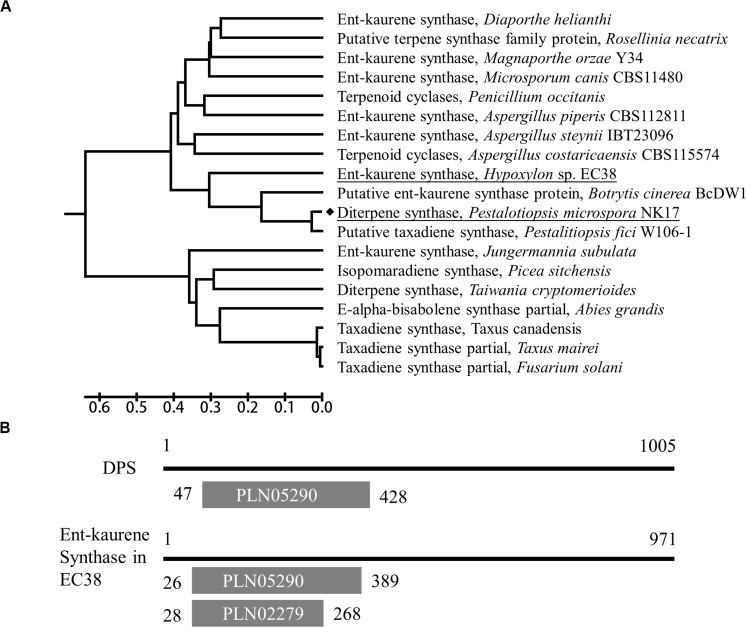
Characterization of the *dps* gene in NK17. **(A)** A UPGMA tree based on the full-length protein sequences of *dps* was built by MEGA7. The *dps* in NK17 is underlined. Diterpene synthases from several other fungi or taxadiene synthase from *Taxus* were used for comparison. **(B)** Protein domain analysis of *dps* compared to Ent-kaurene synthase of *Hypoxylon* sp. EC38. PLN05290, ent-copalyl diphosphate synthetase domain; PLN02279, Ent-kaur-16-ene synthase.

*P. microspora* strain NK17 was isolated by our laboratory and characterized as a producer of a number of dibenzodioxocinones (Niu et al., 2015), with promising application in drug development. A previous study showed that in NK17 there was a diterpene synthase, *dps*, and it is the only enzyme of this kind in the genome (Chen et al., 2015). Intriguingly, this diterpene synthase shows similarity, to some extent, to the taxadiene synthase which catalyzes the first committed step in the biosynthesis of the antitumor drug paclitaxel in Pacific yew and to the bacterial ent-kaurene synthase in cladogram (Figure 1A). To characterize the role of this ubiquitous enzyme in fungi, we employed gene expression information and metabolite profiling comparatively in the wild type and the mutant strain *dps*Δ. We demonstrated that *dps* played a critical role in the biosynthesis of fungal sterols and was indispensable for the growth of *P. microspora* NK17.

## Materials and Methods

### Strains and Plasmids

The fungus *P. microspora* NK17 was isolated by our laboratory and was maintained in potato lactose broth (PLB) at 28°C and 200 rpm or on PLA [PLB with 2% (w/w) agar] at 28°C. The orotidine 5’-phosphate decarboxylase-deficient strain *ura3*Δ was constructed by Chen et al. (2017).

*Escherichia coli* DB3.1 (Invitrogen, Carlsbad, CA, United States) was used to propagate the pOSCAR plasmid (Chen et al., 2017), while the other plasmids used in this study were generally propagated in *E. coli* DH5α. *Agrobacterium tumefaciens* LBA4404 was used for *A. tumefaciens*-mediated transformation (ATMT) of NK17 (Yu et al., 2015). All of the bacterial strains were cultured in Luria–Bertani medium with appropriate antibiotics at 37°C (for *E. coli*) or 28°C (for *A. tumefaciens*) and 200 rpm when needed. An inducing medium (IM) with 200 mg/ml uracil and 20 mg/ml acetosyringone (AS, Sigma, St. Louis, MO, United States) was used as the minimal medium for the selection of NK17 transformants.

### Construction of the Deletion Vector pOSCAR-*dps*Δ

The deletion vector of *dps* was constructed by the in-fusion method. A pOSCAR plasmid containing the *ura3* gene was used as the marker (Chen et al., 2017). First, the 5’-flank and the 3’-flank were amplified from the NK17 genome *via* PCR and purified by a DNA Gel Extraction Kit (Axygen, Union City, CA, United States). Then, the plasmid was cut by the enzyme *Eco*RI, and an in-fusion reaction was performed to add the 5’-flank to pOSCAR, resulting in the intermediate plasmid pOSCAR-*dps*Δ-up. The primer pair *dps*Δ-up-F/ura3-R (Table 1) was used to verify the plasmid. This intermediate plasmid was next cut by enzyme *Hin*dIII, and the 3’-flank was added to the plasmid *via* an in-fusion reaction. The primer pair ura3-F/*dps*Δ-down-R (Table 1) was applied for PCR to verify the final plasmid by the size of the bonds. Thus, the *dps* deletion vector pOSCAR-*dps*Δ was constructed completely.

**TABLE 1:** Primers used in this study.

Primer	Sequence (5′–3′)
aact-S	ATTCCCAGATCGCCACCT
aact-AS	CTACGACCAGTTCCACATG
hmgs-S	TGACCTTCTGCGATGACA
hmgs-AS	AGACGTGCTCCATGTAGG
hmgr-S	GCGTCACCAGTAGTAGTC
hmgr-AS	AAGATTCGTCGTTCCATCA
mk-S	GCAGCGTCAATGGTGTAA
mk-AS	GCCGAGTTCTTGTGAATCT
pmk-S	GGTTACCTGGTGCTGGAT
pmk-AS	GTGGAGATGTAGGTCAAGAC
idi-S	CCGAGATTGATACGACTTC
idi-AS	GTGCCTTGTAGTGAATGC
ggpps-S	ATAATGCTTCTCTGCTTGTC
ggpps-AS	GGATCGAATGGAGTGTATAAC
actin-S	TCGTGACATCAAGGAGAAG
actin-AS	GAAGCGAGAATGGAACCA
*dps*-up-F	AGCTCAAGCTAAGCTTGACAAGTCCAAGCCTGAG
*dps*-up-R	TGGCTAGGACAAGCTTCGAATACATCCAAGAAGTCTC
*dps*-down-F	AACATCAAGGGAATTCTCTAGACATCGTCAGCCTGAAGATAT
*dps*-down-R	ACGCCGAATTGAATTCAAGACATGCACGTTGGTT
*dps*-up-up	CGCCAATGTCAAGTTCCA
ura3-R	ACTGGTAGTGTGGTAGGTA
Q*dps*-F	CCTCATAATCTTGTTCCAGTC
Q*dps*-R	CAATCTTCCTTACGATCTTCC

### Construction of *dps* Deletion Strain *dps*Δ

Ten micrograms of the pOSCAR-*dps*Δ vector was transformed into *A. tumefaciens* LBA4404 by ATMT following the protocol described by Chen et al. (2017) and Wang et al. (2017). Then, *A. tumefaciens* LBA4404 transformants harboring pOSCAR-*dps*Δ were co-cultured with 10^6^ conidia of *ura3*Δ at 28°C on a nitrocellulose filter, which was spread on an IM minimal medium plate. After 2 days, the filter was transferred to a selection medium PLA plate supplemented with kanamycin (100 mg/ml). Another 2 days later, three transformants were transferred to a new PLA plate. Single-spore isolation was applied for every transformant after it had grown for 5 days. Then, the total DNA from each transformant was extracted from mycelium after growing in 100 ml of PLB for 4 days. The primer pair *dps*Δ-up/ura3-R was used to verify the transformants. In addition, the strains were further verified by Southern blot following the protocol of Zhang et al. (2016).

### RNA Preparation, Reverse-Transcription PCR, and qRT-PCR

TRIzol Kit was used to prepare total RNA from lyophilized mycelia after they had grown for 4 days. Then, reverse-transcription PCR (RT-PCR) was performed as described previously by Zhang et al. (2016).

To examine the expression of *dps* in *dps*Δ, two pairs of primers, actin-F/actin-R for the actin-encoding gene *ACT* and Q*dps*-F/Q*dps*-R for *dps*, were designed to amplify the cDNA of NK17 and *dps*Δ by RT-PCR and qRT-PCR. The assay was also performed to examine the expression of MVA genes. The primers used in this assay are listed in Table 1.

### Analysis of Secondary Metabolites

The particular metabolites, dibenzodioxocinones, and some other secondary metabolites of NK17 and *dps*Δ were analyzed by high-performance liquid chromatography (HPLC) and gas chromatography–mass spectrometry (GC–MS), respectively. First, the two strains, NK17 and *dps*Δ, were cultured in 100 ml of PLA medium for 7 days. Then, 1 ml of spore suspension was added to 500 g of sweet potato waste medium for solid fermentation. After 15 days, 500 ml of methyl alcohol was added into the fermentation medium to extract the metabolites. Next, the extracts were collected by filtration and concentrated to approximately 50 ml. Finally, the extracts of NK17 and *dps*Δ were analyzed by HPLC or GC–MS. The HPLC analysis (Agilent 1100, Agilent Technologies, Santa Clara, CA, United States) was conducted at 40°C, using A Kromasil C18 ODS column (4.6 mm × 250 mm, AKZO Nobel, Gland, Switzerland). The mobile phase of HPLC was MeOH/H_2_O (70/30, v/v) at a flow rate of 1 ml/min, and 20 μl of the sample was injected and analyzed at 227 nm. In the GC–MS condition, 1 μl of sample was injected into GC–MS (Bruker 320), using a DB-3 MS (0.25 μm × 0.25 μm × 30 m) column at analyzing temperature of 250°C, scan range, 45–8,000 Da. In this experiment, the sweet potato waste medium without inoculation was analyzed as the negative control.

### Drug and Stress Sensitivity Assay

To test the effect of *dps* on the growth and the drug sensitivity of NK17, 2 and 4 μg/ml of amphotericin (AmB) and 300 and 600 μg/ml of hydroxyurea (HU) were added to the PLA plates. In addition, 0.5 and 1 M NaCl were added to the PLA plate to test the osmotic sensitivity of these mutants. The temperature susceptibility of the *dps*Δ mutant strains was tested at 15, 22, and 28°C. The wild-type NK17 was used as control. Each set of conditions was applied in triplicates, and the growth of the strains in each assay was recorded daily.

## Results

### Characterization of *dps* in NK17

*Via* functional domain alignment, a diterpene synthase gene in NK17, which shared a remarkable similarity (44.66%) to ent-kaurene synthase in *Hypoxylon* sp. EC38 (Wu et al., 2017; Figure 1), was found and thus designated as *dps* (*dps* in *P. microspora* NK17). A phylogenetic tree was built by the software MEGA7 package based on the deduced protein sequence of *dps* and terpene synthase in other fungi (Figure 1A). There is a conserved ent-copalyl diphosphate synthase domain, PLN05290, in the protein (Figure 1B). The sequence similarity suggests that *dps* was potentially responsible for the biosynthesis of diterpenes in NK17.

**FIGURE 2:**
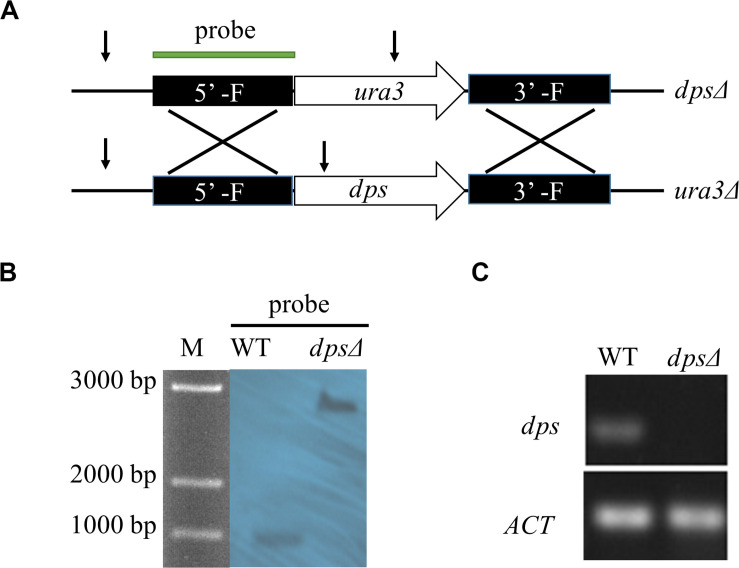
Construction of *dps*-deleted strain *dps*Δ. **(A)** Schematic of the recipient strain *ura3*Δ and *dps*-deleted strain *dps*Δ. In the *dps*Δ strain, *dps* was replaced by a NK17 *ura3* marker through homologous recombination. **(B)** Southern blotting to verify the mutations in *dps*Δ. Genomic DNA from NK17 and *dps*Δ were digested with *Bgl*II [cutting site is shown in panel **(A)** by arrows]. The probe used in Southern blotting is shown in panel **(A)**. When the probe was used, one band of 988 bp was observed in wild-type NK17, and one band of 2,452 bp was observed in both *dps*Δ. **(C)** Transcription of *dps* in wild type and *dps*Δ was detected. No transcription of *dps* was identified in *dps*Δ.

To test the function of *dps*, we created a disruption of *dps* by homologous targeting *via* ATMT in NK17 *ura3*Δ using the selection marker *ura3* complementation (Figure 2A). One of the mutation strains, named *dps*Δ, was selected for Southern blotting. The 5’ flanking region was used as the probe to hybridize to a fragment of 988 bp (in wild type) and a fragment of 2,452 bp in *dps*Δ (Figure 2B). The blotting confirmed the disruption of *dps*. The result of real-time PCR also showed the loss of expression of *dps* in *dps*Δ (Figure 2C).

### Roles of *dps* in the Biosynthesis of Secondary Metabolites

**FIGURE 3:**
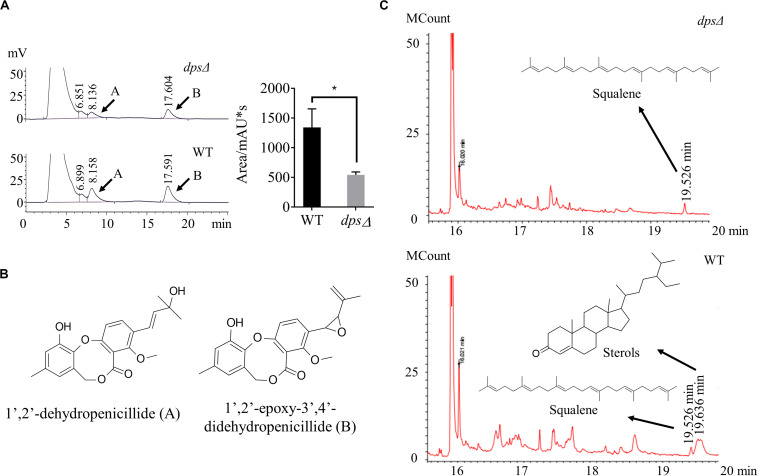
High-performance liquid chromatography (HPLC) and gas chromatography–mass spectrometry (GC–MS) analysis of the secondary metabolites in the mutant strains, *dps*Δ and wild type. **(A,B)** HPLC profiling of the secondary metabolites. The samples were prepared from sweet potato waste fermentation extracts. Compounds **(A,B)** were two dibenzodioxocinones, the main metabolites of NK17, that had been previously identified (Liu Y. et al., 2019). Each condition was done in three independent assays. The peak area of compound **(B)** is shown in the right part of panel **(A)**. *p < 0.05. **(C)** GC–MS profiling of the secondary metabolites in *dps*Δ and NK17. The compound at the retention time of 19.526 min in both *dps*Δ and NK17 was squalene. However, the peak of sterols at 19.636 min was present in NK17 and absent in *dps*Δ.

Diterpene synthase is a key enzyme in the biosynthesis of diterpenes (Koksal et al., 2011; Pelot et al., 2019). To investigate whether *dps* was required for the group of secondary metabolites, we first conducted HPLC profiling for the deletion strain *dps*Δ. Extracts were prepared from sweet potato waste cultures statically grown for 13 days at 28°C. In HPLC profiling, we found that in *dps*Δ strains the production of dibenzodioxocinones was reduced to 37% of the wild-type level (Figure 3A). In a previous study, we have reported that dibenzodioxocinones are the number of polyketides and a dominant group of secondary metabolites in NK17 (Figure 3B; Niu et al., 2015; Liu Y. et al., 2019). In this analysis, dibenzodioxocinone 1’,2’-epoxy-3’,4’-didehydropenicillide (Liu Y. et al., 2019) was monitored at a retention time of 17.6 min. In addition, the HPLC analysis found no new compounds after the deletion of the *dps* gene (Figure 3A).

To better understand the roles of *dps* in secondary metabolism, GC–MS was applied to compare the production of terpenes in the extracts of *dps*Δ and the wild type. As a result, we found that the peak corresponding to sterols, at a retention time of 19.636 min in the total ion current, was absent in the metabolites of *dps*Δ (Figure 3C). Sterols are triterpenoids that are usually cyclized by sterol synthase, such as lanosterol synthase and cycloartenol synthase (Nes, 2011), instead of diterpene synthase. Additionally, in the GC–MS spectrum, the peak corresponding to squalene, the key precursor of sterols (Nes, 2011), was observed at a retention time of 19.526 min in both *dps*Δ and the wild type (Figure 3C and Supplementary Figure S1). These data suggest that *dps* participates in the biosynthesis of precursors of triterpenoids, e.g., sterols, in NK17 and we were unable to detect any new diterpenes in the metabolites of the wild-type NK17.

### *dps* Is Required for the Growth of NK17 and Resistance to External Stress

**FIGURE 4:**
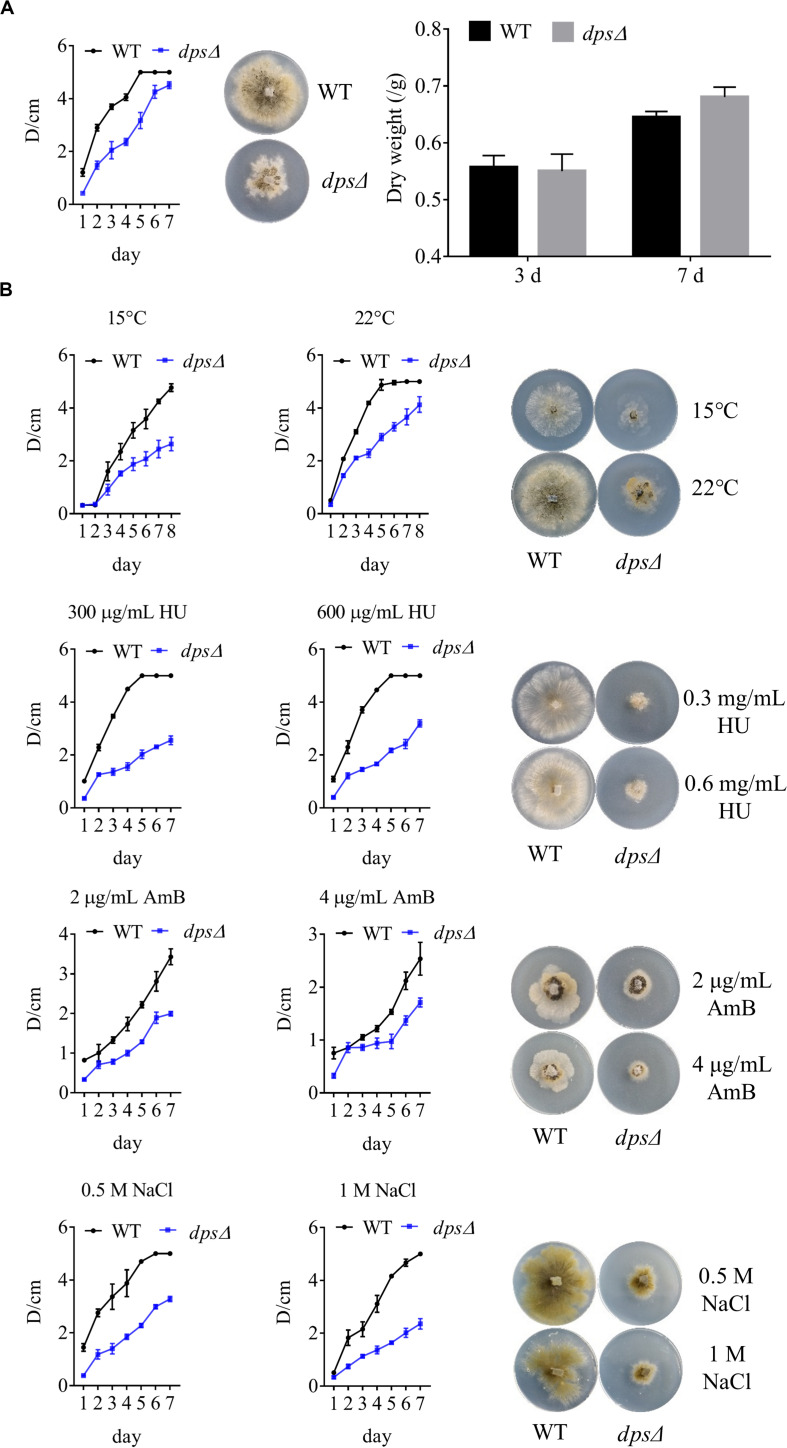
Phenotypic characterization and stress tests of NK17 and *dps*Δ. **(A)** Growth curve and dry weight of mycelia of NK17 and *dps*Δ at 28°C. **(B)** Stress tolerance to different concentrations of HU, NaCl, and AmB and to low temperature on PLB with 2% (w/w) agar (PLA). PLA without any supplement at 28°C **(A)** served as the control. The growth and the development of *dps*Δ were greatly slowed, and the mutant was more sensitive to HU, NaCl, and AmB. In addition, *dps*Δ rarely grew when 2 mg/ml AmB was added, although the growth of the wild type was also slightly slowed under these conditions. Each condition was done in three independent assays. The growing status in the plate on the fourth day is shown.

As components in cellular membranes, sterols play a critical role in the physiology of eukaryotic cells, such as in oxidative stress and in membrane structure (Gold et al., 2016; Liu Z. et al., 2019). We observed, as discussed above, that *dps* could affect the biosynthesis of sterols in NK17 (Figure 3C). To test the role of *dps* in the growth of NK17, we further analyzed the colony diameters of *dps*Δ and the wild type on the PLA plates at 28°C (Figure 4A) and lower temperature, i.e., 15 and 22°C (Figure 4B). The colony diameter of *dps*Δ was nearly 50% smaller than that of the wild type (Figure 4A). These results clearly demonstrate that *dps* plays an important role in the growth of NK17.

Furthermore, to observe the function of *dps* against external stress, different concentrations of sodium chloride (NaCl), AmB, and HU were added to the culture (Figure 4B). NaCl is an osmotic reagent that can regulate the osmotic pressure in the cell (Wang et al., 2017), and AmB and HU were added to detect resistance to antifungal fungicides. Treatment with 1 M NaCl or 600 μg/ml HU led to colonies with the diameters of *dps*Δ only at 35% that of the wild type (Figure 4B). In addition, *dps*Δ was more sensitive than the wild type to AmB and rarely grew in the presence of 2 μg/ml AmB (Figure 4B). The data demonstrated that the growth status of *dps*Δ worsened under external stress, which may be due to a disruption of the sterol biosynthesis pathway (Kim and London, 2015; Gold et al., 2016).

### Phenotypic Defects of *dps*Δ Are Restorable by the Addition of Exogenous Sterols

**FIGURE 5:**
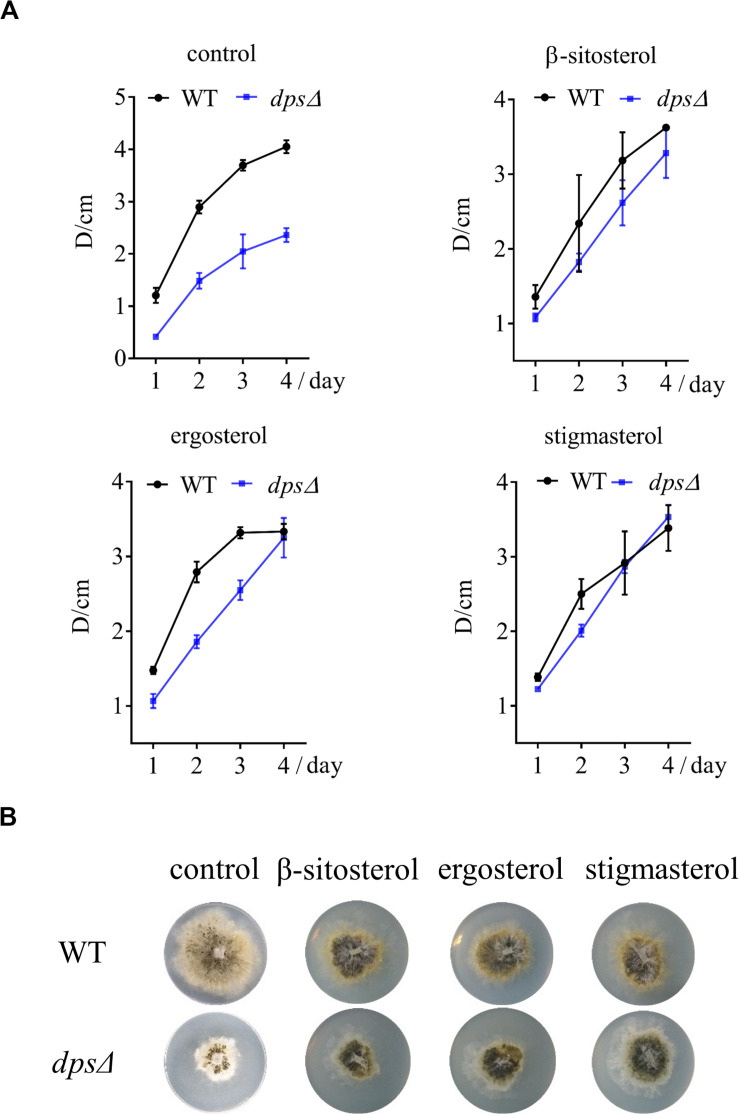
The growth of *dps*Δ was recovered by the exogenously supplied sterols to the culture. **(A)** Growth curve of NK17 and *dps*Δ stigmasterol, β-sitosterol, and ergosterol were added separately at 5 mg/L to recover the growth of *dps*Δ. **(B)** After 4 days of culturing, the growth and the development of *dps*Δ were recovered. In addition, the growth of NK17 was slightly slowed when sterol was added. Each condition was done in three independent assays.

Because deleting *dps* affected the synthesis of sterols, which may further inhibit the growth of NK17, we added three sterols, β-sitosterol, stigmasterol, and ergosterol, to a final concentration of 5 mg/L on the PLA plates. As expected, all the sterols restored the growth of *dps*Δ (Figure 5A), declaring the direct relation between the biosynthesis of sterols and fungal growth. Besides that, we noticed that the colony diameter of *dps*Δ was a little larger than that of the wild type after the addition of stigmasterol after culturing for 3 days (Figures 5A,B). Therefore, it was very likely that the exogenous stigmasterol generated an inhibitory effect in the wild-type NK17 strain.

### Disruption of *dps* Affects the Expression of MVA Pathway Genes

**FIGURE 6:**
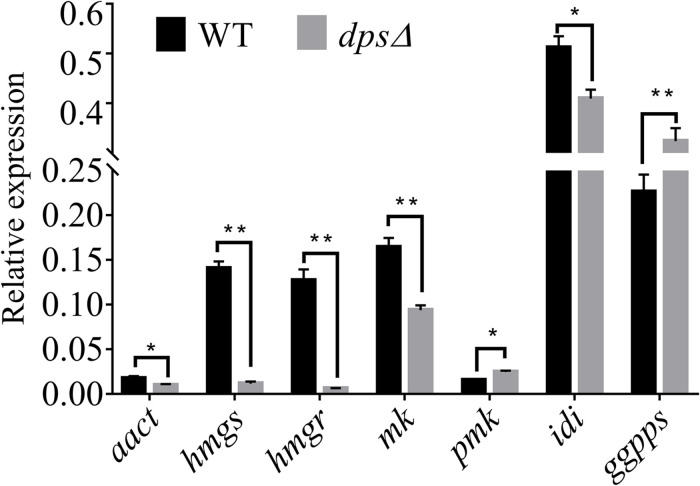
Expression of genes in the mevalonate (MVA) pathway in wild type and *dps*Δ. The expression of the MVA pathway genes in NK17 and *dps*Δ were detected by qRT-PCR. The values are expressed as a relative expression with respect to the gene for actin as an endogenous control. *aact*, acetyl-CoA C-acetyltransferase gene; *hmgs*, 3-hydroxy-3-methylglutaryl CoA synthase gene; *hmgr*, 3-hydroxy-3-methylglutaryl CoA reductase gene; *mk*, mevalonate kinase gene; *pmk*, phosphomevalonate kinase gene; *idi*, isopentenyl diphosphate isomerase gene; *ggpps*, geranylgeranyl diphosphate synthase gene. *p < 0.05, **p < 0.01.

A previous study demonstrated that the biosynthesis of sterols started with IPP and DMAPP, two C5 isoprene units synthesized by the MVA pathway (Engels et al., 2008; Cha et al., 2012; Bian et al., 2017). Total genome sequencing and mRNA sequencing of NK17 were performed to identify the MVA pathway genes in NK17 *via* protein sequence BLASTP on NCBI. All seven genes in the MVA pathway could be found and the transcriptome sequencing (mRNA-seq) of NK17 suggested that these genes (*aact*, *hmgs*, *hmgr*, *mk*, *pmk*, *idi*, and *ggpps*) in the MVA pathway could be constitutively expressed (Table 2). Real-time PCR amplification was set to confirm the RNA sequencing data (Figure 6). To detect whether disrupting *dps* could affect the expression of the MVA pathway, qRT-PCR was applied to analyze the expression of the seven genes in the pathway. Five genes of the seven, *aact*, *hmgs*, *hmgr*, *mk*, and *idi*, had a significant lower expression in *dps*Δ compared to the wild type (Figure 6), and among them, *hmgs* (8.3% of the wild-type level in *dps*Δ) and *hmgr* (4.9% in *dps*Δ) had the largest decrease in expression (Figure 6). Interestingly, the expression of the other two genes, *pmk* and *ggpps*, turned out to be activated instead by more than 60 and 30%, respectively (Figure 6).

**TABLE 2:** Identification and RNA-seq analysis of *dps* and genes in MVA pathway.

Gene	Size/bp	Blastp homologue	Identity (100%)	E value	FPKM^a^
*aact*	1,296/431	*Pestalotiopsis fici* W106-1	96.29	0.0	86.61
*hmgs*	1,386/461	*Pestalotiopsis fici* W106-1	93.49	0.0	111.38
*hmgr*	3,510/1,169	*Pestalotiopsis fici* W106-1	95.63	0.0	42.59
*mk*	1,569/522	*Colletotrichum trifolii*	71.37	0.0	61.65
*pmk*	1,173/390	*Hypoxylon* sp. EC38	57.21	4e−164	22.81
*idi*	765/254	*Pestalotiopsis fici* w106-1	96.85	0.0	203.83
*ggpps*	1,203/400	*Pestalotiopsis fici* W106-1	93.98	0.0	112.73
*dps*	3,015/1,004	*Hypoxylon* sp. EC38	44.66	0.0	8.19

*^*a*^Fragment per kilobase of exon model per million of mapped fragments.*

## Discussion

*P. microspora* is an endophytic fungus that is well known for producing a great number of secondary chemicals, including paclitaxel (Strobel et al., 1996). However, it seems that the fungus in our lab lost its capability to produce paclitaxel after extended subculture in the laboratory, although some terpenoids and their derivatives were identified from solid fermentation metabolites by GC–MS, i.e., squalene and sterols (Figure 3C). The biosynthesis of sterols and paclitaxel relied on the common precursors, IPP and DMAPP, the two C5 units (Dhingra and Cramer, 2017). By far, the best-studied biosynthesis pathways of these two C5 units were the methyl-D-erythritol 4-phosphate pathway and the MVA pathway (Nes, 2011). Isotope labeling method in plants and microorganisms demonstrated that both of these two routes existed in higher plants, while in fungi, the biosynthesis of IPP and DMAPP mainly depended on the MVA pathway (Lichtenthaler, 1999; Masse et al., 2004). In this report, we defined the upstream pathway that putatively leads to the biosynthesis of diterpenoids, i.e., the MVA pathway for terpenoid biosynthesis (Engels et al., 2008). As a result, all seven genes in this pathway were determined to be constitutively expressed (Figure 6). The identification of the sterols, which were biosynthesized from squalene, another polymer of IPP and DMAPP (Dhingra and Cramer, 2017), by GC–MS verified this conclusion.

The diterpene synthase in fungi has been well studied to increase the production of diterpenoids or to synthesize new diterpenoids *via* heterologous expression (Driller et al., 2019). However, there was no report about the function of diterpene synthase on the biosynthesis of triterpenes. By genome sequencing, only one copy of the gene encoding a diterpene synthase was found in NK17, which was initially speculated to be involved in the biosynthesis of paclitaxel. *Via* ATMT, the diterpene synthase, *dps*, was disrupted for the purpose of defining its function in NK17 in our study (Figure 2). Interestingly, the peaks of sterols in the GC–MS spectrum were absent in the deletion mutant (Figure 3C). A wide peak, a rather sharp peak, at 19.636 min in the GC–MS spectrum demonstrated that NK17 could produce more than one sterol. Additionally, many sterols could be matched with high scores of R. Match and F. Match by comparing the molecular fragments (Supplementary Figure S2). These findings, together with the changes of MVA pathway gene expression in *dps*Δ (Figure 6) and the appearance of squalene in the GC–MS spectrum (Supplementary Figure S1), suggest that *dps* participates in the biosynthesis of sterols.

Sterols have been extensively studied because of their important roles in drug resistance, developmental regulation, cell signaling, and cell growth. Therefore, a large number of sterols isolated from bacteria, fungi, and plants have been reported and researched (Kim and London, 2015; Lamberson et al., 2017; Rivas-Marin et al., 2019; Ruan et al., 2019). Some sterols have even been implicated in human diseases, including Alzheimer’s disease and cancers (Liu Z. et al., 2019). The disruption of *dps* in NK17 resulted in increased sensitivity to high osmotic stress and antifungals and decreased growth (Figures 4A,B), which could be improved by the addition of sterols to the medium (Figure 5). In addition, we also noticed that the colony diameters of NK17 with exogenous sterols were smaller than those of the wild type without sterols (Figure 5), demonstrating that the oversupplied sterols in the media could significantly suppress the growth of NK17, in consistence with the result that sterols were particularly important for several cellular processes in fungi and would become a risk factor regardless of whether there was an excess or a shortage (Liu Z. et al., 2019). These data also reinforce the point that the *dps* of NK17 participated in the biosynthesis of sterols, although the synthesis of squalene was rarely affected (Liu Z. et al., 2019). In addition, the growth of NK17 was apparently slower when *dps* was knocked out (Figure 4A), which likely is the result from the impaired biosynthesis of sterol production (Gold et al., 2016; Liu Z. et al., 2019). However, when we measured the biomass of the wild type and *dps*Δ in PLB, there was hardly a measurable difference between these two strains (Figure 4A). This result demonstrates the fact that there was a great difference between solid and liquid cultures of filamentous fungi in many aspects, for instance, the growth rate, gene expression, colonial morphology, and secondary metabolite biosynthesis (Bigelis et al., 2006). Thus, our data showed that DPS was only indispensable when the fungus was grown on solid plate such as PLA.

In conclusion, by disrupting the diterpene synthase (*dps*) in NK17, we revealed for the first time that this protein has two functions. One is that *dps* affects the biosynthesis of triterpenes, such as sterols, although the role, if any, of *dps* in the biosynthesis of paclitaxel is still uncertain. The other role of the enzyme is that *dps* is required for the growth and the development of fungi by affecting the biosynthesis of sterols.

## Data Availability Statement

All datasets generated for this study are included in the article/Supplementary Material.

## Author Contributions

XZ and YLiu conceived and designed the study. YLiu, AD, LC, DW, QX, BX, and YLin performed the experiment. LC and XH provided the plasmids. YLiu and XZ wrote the manuscript. QX and XZ reviewed and edited the manuscript. All the authors read and approved the manuscript.

## Conflict of Interest

LC was employed by Zhejiang Medicine Co., Ltd., Zhejiang, China. The remaining authors declare that the research was conducted in the absence of any commercial or financial relationships that could be construed as a potential conflict of interest.
