# Supplementary material for: A Fungal Diterpene Synthase Is Responsible for Sterol Biosynthesis for Growth
Source: Front Microbiol. 2020 Jul 10;11:1426. doi: 10.3389/fmicb.2020.01426 (PMC7365874; doi:10.3389/fmicb.2020.01426)
Supplement: Supplementary file 1 [file Data_Sheet_1.PDF]

Supplementary Material

A fungal diterpene synthase is responsible for sterol biosynthesis for growth

Yanjie Liu[1], Anqing Duan[1], Longfei Chen[2,3], Dan Wang[3], Qiaohong Xie[1], Biyun Xiang[1], Yamin Lin[1], Xiaoran Hao[1], Xudong Zhu[1*]

[1]Beijing Key Laboratory of Genetic Engineering Drug and Biotechnology, Institute of Biochemistry and Biotechnology, College of Life Sciences, Beijing Normal University, Beijing, 100875, China

[2]Zhejiang Medicine Co., Ltd., Zhejiang, China

[3]Department of Microbiology, College of Life Sciences, Nankai University, Tianjin, 300071, China

*** Correspondence:**

Xudong Zhu

Tel./fax: +86 010 58804266

zhu11187@bnu.edu.cn

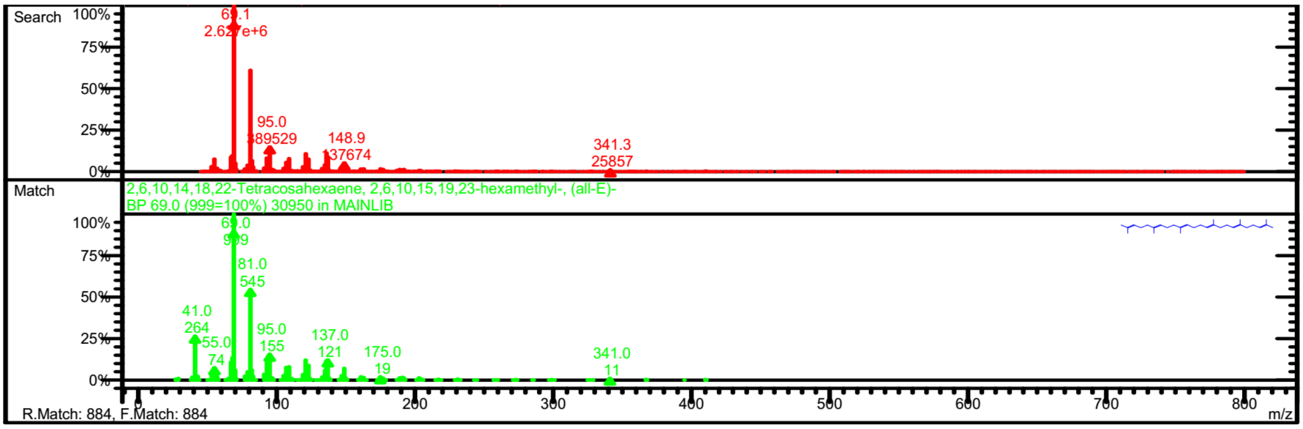

Figure S1 Molecular match of squalene in GC-MS.

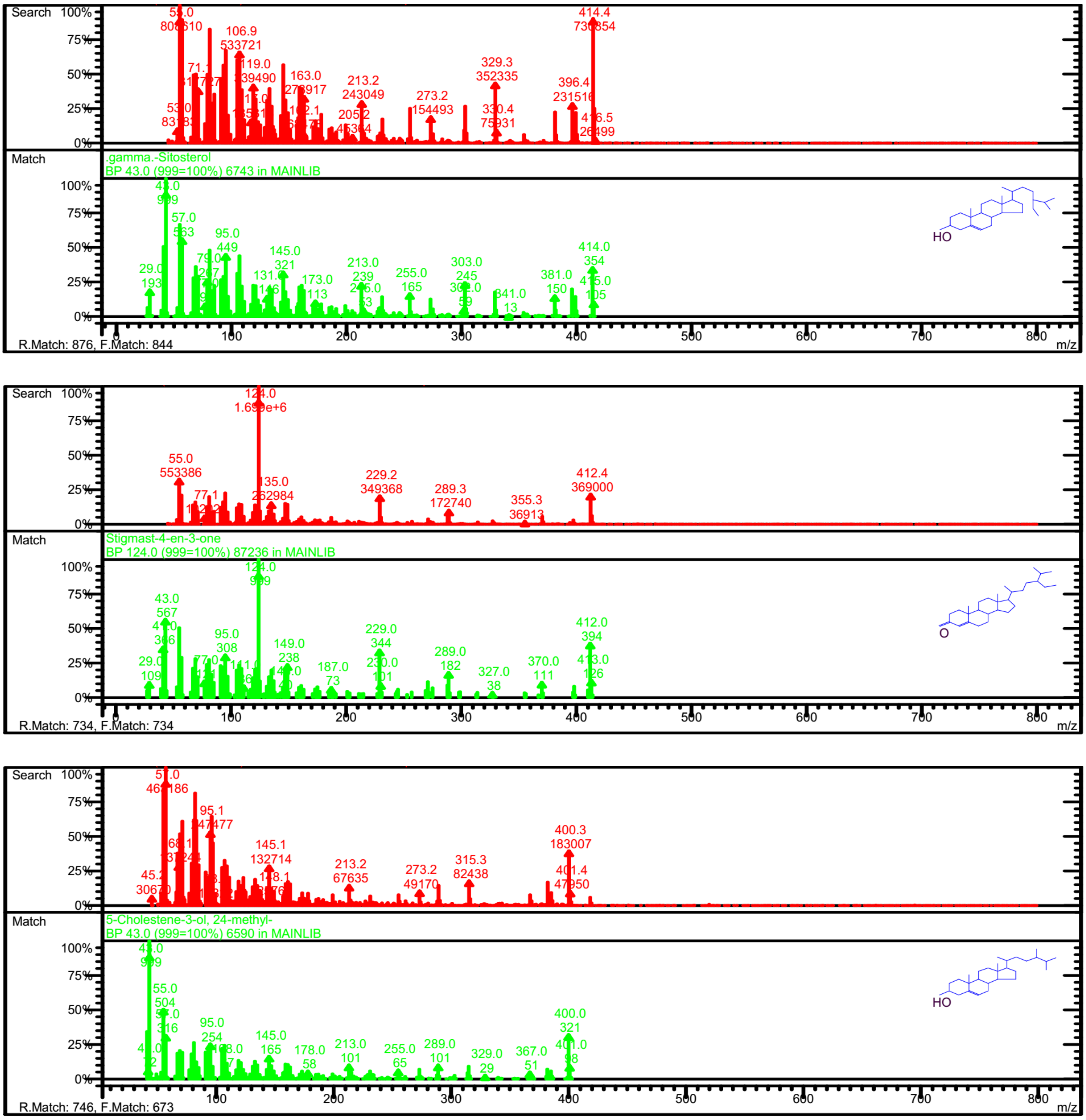

Figure S2 Molecular match of sterols in GC-MS.
